# mTOR Pathway Expression as Potential Predictive Biomarker in Patients with Advanced Neuroendocrine Tumors Treated with Everolimus

**DOI:** 10.3390/cancers12051201

**Published:** 2020-05-10

**Authors:** Fabio Gelsomino, Andrea Casadei-Gardini, Francesco Caputo, Giulio Rossi, Federica Bertolini, Tiziana Petrachi, Andrea Spallanzani, Elisa Pettorelli, Shaniko Kaleci, Gabriele Luppi

**Affiliations:** 1 Department of Oncology and Hematology, University Hospital of Modena, 41124 Modena, Italy; andrea.casadeigardini@unimore.it (A.C.-G.); francesco1990.caputo@libero.it (F.C.); bertolini.federica@aou.mo.it (F.B.); andrea.spallanzani@gmail.com (A.S.); elisa.pettorelli@unimore.it (E.P.); luppi.gabriele@aou.mo.it (G.L.); 2 Pathology Unit, Azienda USL della Romagna, 48121 Ravenna, Italy; giurossi68@gmail.com; 3 Scientific and Technological Park of Medicine “Mario Veronesi”, 41037 Mirandola (MO), Italy; tiziana.petrachi@tpm.bio; 4 Department of Diagnostic Medicine, Clinical and Public Health, University Hospital of Modena, 41124 Modena, Italy; shaniko_k@hotmail.com

**Keywords:** neuroendocrine tumors, everolimus, mTOR inhibitor, survival prediction, prognosis

## Abstract

Background. Everolimus (Eve), which is a mammalian target of Rapamicin (mTOR) inhibitor, is part of the therapeutic armamentarium of neuroendocrine tumors (NETs). Currently, there are no validated biomarkers predicting Eve efficacy in NETs. In this study, we explore whether the expression of phosphorilated (p)-mTOR and p70S6-kinase (S6K), a downstream effector of mTOR, correlates with the outcome of patients with NET that were treated with Eve. Methods. Tissue specimens that were derived from NETs treated with Eve at our Institution were examined for the expression levels of p-mTOR and p-S6K by immunohistochemistry. Response rate (RR), progression-free survival (PFS), and overall survival (OS) were analyzed in two groups: p-mTOR/p-S6K positive (group 1) and p-mTOR/p-S6K negative (group 2). Univariate and multivariate Cox regression analysis were performed. Results. Twenty-four patients with advanced NETs that were treated with Eve were included in the analysis. Eight out 24 (33.3%) patients were both p-mTOR and p-S6K positive. A better median PFS and OS in group 1 (18.2 and 39.9 months) as compared to group 2 (13 and 32.4 months) was depicted, with a toxicity profile that was comparable with data literature. Conclusions. Our study suggests that the activation of mTOR pathway can predict better outcomes in patients with NET treated with Eve. However, these results warrant further confirmation in a prospective setting.

## 1. Introduction

Neuroendocrine neoplasms (NENs) are rare tumors with increasing incidence and prevalence. They are a heterogeneous group of tumors of various origins, which are characterized by their ability to secrete peptides, resulting in distinctive hormonal syndromes. More than 50% of cases of NEN originate in the gastrointestinal system or pancreas and patients commonly present with metastatic disease at diagnosis [1]. Tumor behavior and patient survival depend on a number of factors, including primary site, tumor histology, proliferative index Ki-67, and staging [2,3,4,5,6]. According to 2019 WHO classification, based upon the Ki-67 index and morphology, gastro-entero-pancreatic (GEP) NENs are subdivided into four categories: well-differentiated G1 neuroendocrine tumors, with a Ki-67 index below 3%, G2 neuroendocrine tumors, with a K-67 between 3% and 20%, G3 neuroendocrine tumors, with a well-differentiated morphology and a Ki-67 index above 20%, and poorly differentiated neuroendocrine carcinomas (NECs), with a poorly differentiated morphology and a Ki-67 above 20% [7]. Instead, pulmonary NENs are classified according to the widely agreed World Health Organization (WHO) scheme into: typical carcinoids, atypical carcinoids, large cell and small cell lung carcinomas [8]. While surgery remains the mainstay of treatment with the potential goal of cure in localized disease, treatment of advanced disease, with a number of options now available, requires a coordinated multidisciplinary approach in order to define the best strategy for every patient [9]. Eve, which is an oral inhibitor of m-TOR, binds the cyclophilin FK506 binding protein-12 (FKBP-12) and this complex binds the serin/threonine kinase mammalian target of Rapamicin (mTOR) when it is associated with raptor and mammalian lethal with SEC13 protein 8 (mLST-8) to form a complex, mammalian target of Rapamicin Complex 1 (mTORC1), which inhibits downstream signaling. mTORC1 controls translation, suppresses autophagy, and regulates the transcription and response to DNA damage. mTOR exists in a second complex, mammalian target of Rapamicin Complex 2 (mTORC2), associated with rictor, mammalian stress-activated protein kinase interacting protein 1 (mSin1) and mLST-8, which phosphorylates Akt at Ser-473, leading to full activation of Akt. The major substrates for mTORC1 are ribosomal protein S6 kinase 1 (S6K1) and eukaryotic initiation factor 4E-binding protein 1 (4E-BP1). S6K1 negatively regulates insulin growth factor 1 (IGF-1) receptor signaling through the phosphorylation of insulin receptor substrate 1 (IRS-1), which leads to its proteasomal degradation. The phosphorylation of 4E-BP proteins facilitates the formation of the translation initiation complex that is required for efficient translation of cell cycle regulators (Cyclin D1, ornithine decarboxylase) and transcription factors, such as myelocytomatosis oncogene cellular homolog (Myc) and Hypoxia inducible factor 1α (HIF-1α) [10]. Eve demonstrated antitumor activity in preclinical and clinical phase II and III studies and regulatory agencies have already approved it for the treatment of patients with advanced, progressive, well-, or moderately-differentiated pancreatic neuroendocrine tumors (pNETs) [11,12,13,14] and, more recently, for patients with progressive lung or gastrointestinal neuroendocrine tumors (NETs) [15]. The expression of mTOR pathway components in NENs has been evaluated in few studies and, although with conflicting results, seems to correlate with adverse clinical outcomes [16,17,18,19]. Moreover, little is known regarding the predictive role of mTOR pathway expression of Eve efficacy in NENs [20,21]. The aim of the study is to explore whether the expression of phospho(p)-mTOR and phosphor(p)-S6K correlates with the outcome of patients with NETs of various origins treated with Eve.

## 2. Materials and Methods

### 2.1. Study Population

Tumor tissue blocks from patients with a diagnosis of NET treated with Eve at University Hospital of Modena within different clinical trials (RADIANT, EUDRACT number 2006-001247-64, Ethics Committee approval number 2708; RADIANT-2, EUDRACT number 2006-004507-18, Ethics Committee approval number 701; RADIANT-3, EUDRACT number 2006-006810-75, Ethics Committee approval number 2270; COOPERATE-2, EUDRACT number 2010-023183-40, Ethics Committee approval number 3104) or expanded access program between October 2006 and March 2013 were analyzed. All of the samples were collected before treatment with Eve.

Demographic and clinical informations were extracted from patients’ medical records. Informed consent for the treatment of patient’s data was obtained for all patients that were treated at our institution, in accordance with local and international law.

### 2.2. Immunohistochemistry

Four-micron thick sections from a representative formalin-fixed, paraffin-embedded tumor specimen were obtained for immunohistochemical analysis. Briefly, after deparaffinization, rehydration, and heat-based antigen retrieval, the slides were incubated with the following primary antibodies: monoclonal mouse anti Ki67 (MIB-1, Ventana/Roche, Tucson, AZ), monoclonal mouse anti chromogranin A (DAK-A3, Dako, Glostrup, Denmark), polyclonal rabbit anti-synaptophysin (Ventana/Roche, Tucson, AZ), monoclonal mouse anti CD56 (123C3, NeoMarkers, San Ramon, CA), monoclonal rabbit anti phospho-mTOR (clone p-mTOR49F9, Cell Signaling Technology, Danvers, MA, USA), and monoclonal mouse anti phospho-p70S6-kinase (clone p70S6-kinase 1A5, Cell Signaling Technology, Danvers, MA, USA).

Immunohistochemistry reactions were performed while using an automated immunostainer (ULTRABenchmark, Ventana, Tucson, AZ, USA) and 3’-3-diaminobenzidine (DAB) was used as chromogene. All of the slides were counterstained with Carazzi’s haematoxylin. Neuroendocrine intestinal cells and nervous structures were used as internal positive control for neuroendocrine proteins. Sections of a pulmonary typical carcinoid known to be positive for p-mTOR and p-p70S6-kinase were used as external positive control for phospho-mTOR and p-S6-kinase protein expression. All of the determinations were also validated while using parallel control sections omitting the primary antibodies in each immunohistochemistry batch.

### 2.3. Evaluation of Staining of Tissue Slides

The immunohistochemical results were reviewed and recorded by a pathologist blinded to clinical outcome data. In particular, staining intensity was scored as 0 (no immunostaining), 1+ (weak), 2+ (moderate), and 3+ (strong). Positivity was quoted when at least 10% of tumor cells reacted with at least a moderate intensity (2+) staining in the adequate sub-cellular localization (cytoplasmic for p-mTOR and nuclear for phospho-p70S6K).

### 2.4. Statistical Analysis

Response rate (RR), progression-free survival (PFS), and overall survival (OS) were analyzed in both groups. RR was defined according to response evaluation criteria in solid tumors (RECIST) criteria. PFS was defined as the time from the start of therapy with Eve and the progression of the disease or death, while OS was defined as the time from the start of therapy and death from any cause. Stata was used for all statistical analyses (Stata Statistical Software, Release 12 [2011]; StataCorp LP, Lakeway Drive, Texas, USA). The difference between the categorical variables was calculated using Fisher’s exact test, while Student’s t-test was used for continuous variables. PFS and OS distributions were estimated while using the Kaplan–Meier method and study groups were compared using log-rank tests. The results were considered to be statistically significant at a level of *p* < 0.05. Univariate and multivariate Cox regression analysis (adjusted for age, site of origin, and grading) were performed.

## 3. Results

### 3.1. Clinical-Pathological Characteristics

**Figure 1:**
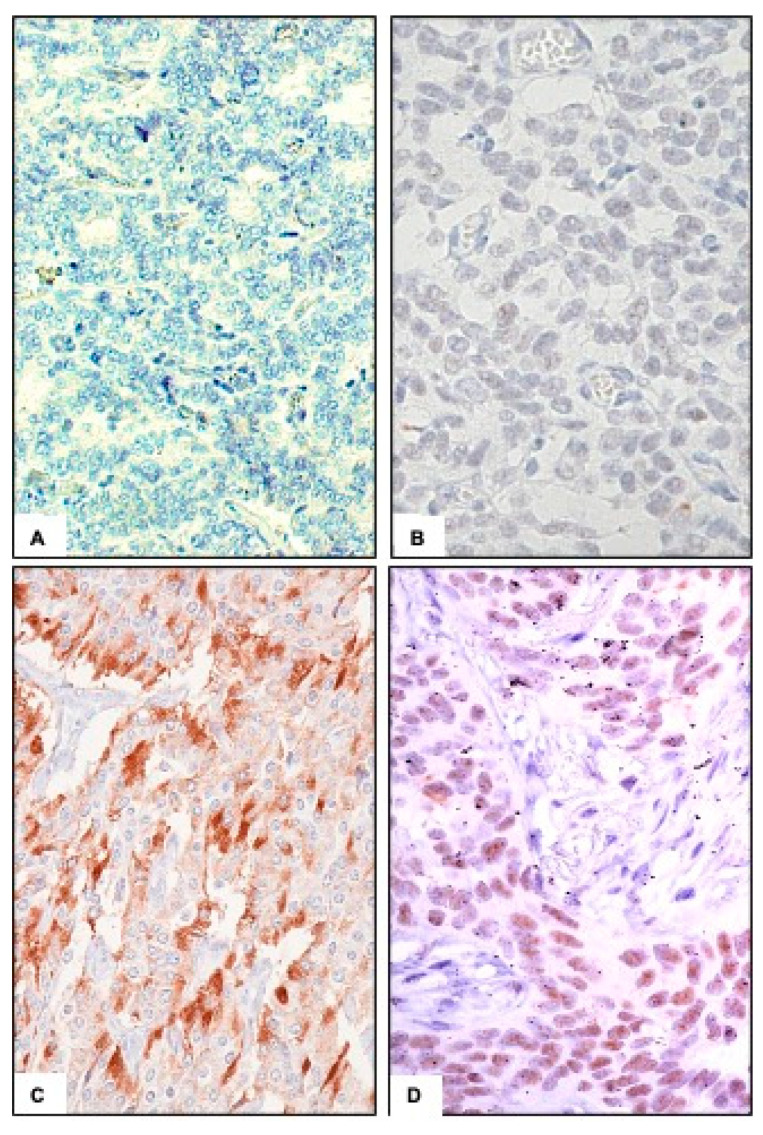
Immunohistochemical diffuse staining of p-mammalian target of Rapamicin (p-mTOR) (**C**) and p-S6K (**D**) in a case of neuroendocrine tumors (NET) of the ileum (200-fold magnification). Negative controls are shown in (**A**) and (**B**).

We analyzed 24 patients with advanced NET of various origin treated at our Institution. Table 1 lists patients’ characteristics. Respectively, eight out 24 patients were p-mTOR and p-S6K positive (Figure 1c,d) and 16 were negative for both, with a concordance rate between p-mTOR and p-S6K expression of 100%. In 14 patients (58.3%), the specimen derived from a metastatic site, in eight patients (33.3%) from the primary tumor, while in two patients (8.3%), the analysis was performed on the primary tumor and confirmed on a metastatic site.

The median age at diagnosis was 59.3 (range 28–84). All of the patients had progressive disease before starting Eve. 14 patients (58.3%) had a pNET, six (25%) had an ileal NET and four patients (16.7%) had a NET of other origin (two bronchial carcinoids, one thymic, and one of unknown origin). Nine patients (37.5%) had a well-differentiated (G1) NET, 14 patients (58.3%) had a moderately-differentiated (G2) NET, and only one patient (4.2%) had a NEC, according to the WHO 2019 classification. None of the patients had a G3 NET. All the patients in group 1 had a G1 NET, while in group 2 14 patients (87.5%) had a G2 NET, one patient had a G1 NET and another a NEC. 13 patients (54.2%) had the primary tumor resected, seven out eight (87.5%) in group 1 and six out 16 (37.5%) in group 2. The median interval between the diagnosis of advanced disease and the start of Eve therapy was 53.7 months, longer in group 1 (106.75 months) than in group 2 (27.2 months). The median number of lines of therapy prior to Eve treatment in group 1 and 2 was 3 and 2.1, respectively, while only three patients (12.5%) were treatmentnaive. In 20 cases (83.3%) Eve was combined with a somatostatin analog (SSA).

### 3.2. Response Rate, Progression-Free and Overall Survival

**Figure 2:**
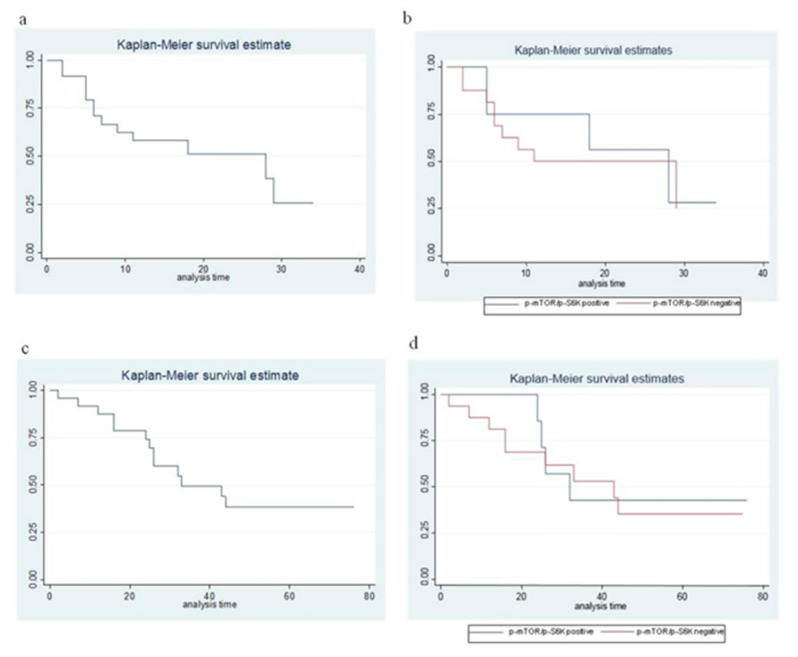
Progression-free and overall survival in the whole series (**a**,**c**) and according to p-mTOR/p-S6K expression (**b**,**d**).

**Figure 3:**
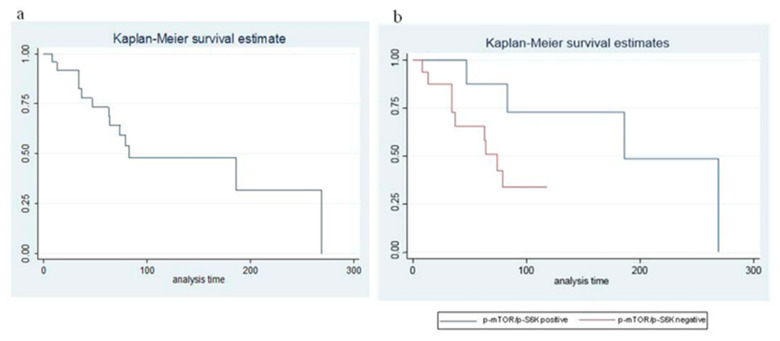
Survival from diagnosis of advanced disease in the whole series (**a**) and according to p-mTOR/p-S6K expression (**b**).

Objective response was only evaluable in 22 out 24 patients (in one case for withdrawal of the informed consent before the first radiological evaluation, in the other case because the patient was treated with transarterial chemoembolization (TACE) while he was receiving Eve; therefore, the objective response obtained cannot be unequivocally attributed to Eve). Both of the non-evaluable patients were in group 1. Of the 22 evaluable patients, 3 (13.6%) obtained a partial response (1 in group 1 and 2 in group 2), 17 (77.3%) had a stable disease and 2 (9.1%) had a disease progression as best response. The median PFS was 14.7 months (Figure 2a), 18.2 in group 1 and 13 months in group 2 (*p* = 0.62), respectively (Figure 2b). Median OS was 34.9 months (Figure 2c), 39.9 in group 1, and 32.4 months in group 2 (*p* = 0.74), respectively (Figure 2d). After a median follow up of 90 months, median survival from diagnosis of advanced disease was 88.4 months (Figure 3a), 148.25 in group 1, and 58.5 in group 2 (*p* < 0.001), respectively (Figure 3b). In patients with pNET, the median survival from diagnosis of advanced disease was 57 months, while, in patients with ileal, NET was 155.5 months. The toxicity profile was comparable with the literature data. Univariate and multivariate Cox regression analysis (adjusted for age, site of origin, and grading) showed no statistically significant differences in terms of PFS, OS, and OS from the diagnosis of advanced disease between the two groups (Table 2 and Table 3).

## 4. Discussion

Since we are moving towards the era of personalized medicine, it is more relevant to have predictive rather than prognostic biomarkers, in order to improve the outcome of the molecularly selected patients, thus avoiding unnecessary toxicities and providing a better allocation of economic resources. As regards the prognostic role, it is plausible that the activation of mTOR pathway portends a more aggressive phenotype with a worse prognosis, although the literature results are conflicting [16,17,18,19]. In a paper by Missiaglia and colleagues, 72 patients with pNET were analyzed and low expression of tuberous sclerosis 2 (TSC2) and phosphatase and tensin homolog (PTEN), whose deregulation activates the mTOR pathway, was significantly associated with shorter disease-free and overall survival [16]. Moreover, Qian and co-workers examined 195 NETs of various origins and demonstrated an association between the expression of mTOR and some downstream targets and adverse clinical outcomes [22]. However, associations between the mTOR pathway component expression and clinical outcomes in other studies of NETs have been less definitive [18,19]. Regarding the predictive role, very few studies have evaluated the correlation between mTOR pathway components expression and mTOR inhibitors efficacy. Gagliano and colleagues demonstrated that NVP-BEZ235, a novel dual inhibitor of Phosphatidylinositol 3-kinase (PI3K) and mTOR, is twice as potent as Eve in reducing cell viability and activating apoptosis in human bronchial carcinoid primary cultures. Resistant cells display lower levels of mTOR and other downstream targets, indicating that these proteins may be useful as predictive markers of resistance to mTOR and PI3K/m-TOR inhibitors [20]. Moreover, Duran and colleagues, in a group of 37 NECs that were treated with temsirolimus, demonstrated that higher basal level of p-mTOR are predictive of a better response and an increase of pAKT (an upstream protein of mTOR pathway) levels and a decrease of p-mTOR levels after two weeks of treatment seem to be predictive of a longer time to progression [21].

In our work, patients with a p-mTOR/p-S6K positive tumor seem to have a better PFS and OS as compared to those with p-mTOR/p-S6K negative tumor. Our series, consistently with that of Qian and colleagues, show that the expression of p-mTOR and p-S6K is more frequent in patients with ileal NETs [22], while, in another series, the expression of p-mTOR is more frequent in foregut than in midgut NETs [17]. However, the association between p-mTOR and p-S6K with a low Ki-67 labeling index and a low grading is what differentiated our series from the others, and this might probably be due to the low number of patients or other unknown factors. In fact, in the case series of Qian and Kasajima, the expression of p-mTOR and other components of the pathway seems to be associated with a higher proliferative rate and worse outcomes, while, in our series, all patients with p-mTOR/p-S6K positive tumors have a G1 NET, more frequently with an ileal primary and have a better survival from the diagnosis of advanced disease when compared to p-mTOR/p-S6K negative, although the difference does not reach statistical significance in both univariate and multivariate analyses, probably due to the low sample size. Thus, we cannot exclude that the difference between the two groups might be due to the more favorable prognostic characteristics of the p-mTOR/p-S6K positive tumors, rather than the higher efficacy of Eve, which is further highlighted by the longer median interval from the diagnosis of advanced disease and the start of therapy with Eve in this group. Another significant difference between the two groups is the higher frequency of primary tumor resection in the p-mTOR/p-S6K positive group, which seems to correlate with a better survival in retrospective series [23]. This difference could be explained by the higher percentage of intestinal NET in this group, in which the resection of the primary tumor, even when asymptomatic, is often recommended, in order to avoid complications, such as mesenteric fibrosis, intestinal ischemia, and occlusion. We performed the analyses on a metastatic site or the primary tumor, whichever was available, but in the only two cases in which we had both tissues, the results were concordant.

Furthermore, although the majority of NETs are sporadic tumors, a proportion of them (10–15%) are attributable to familial cancer syndrome, like Multiple Endocrine Neoplasia (MEN) 1 and 2, von-Hippler-Lindau Syndrome and Tuberous Sclerosis Complex 1 (TSC-1) and Tuberous Sclerosis Complex 2 (TSC-2) [24]. TSC is a multisystem, autosomal dominant condition, which might present as a single, index case or familial disorder, caused by germline mutations in the TSC1 or TSC2 tumor suppressor genes, which encode for the hamartin and tuberin proteins, respectively [25]. The TSC-1 and TSC-2 proteins form a complex and function as tumor suppressors by inhibiting mTORC1 kinase [25,26]. Many findings suggest that patients with TSC who have mutations of the TSC genes, which are components of the AKT/mTOR oncogenic cascade, are predisposed to develop pNETs [27]. Although none of the patients that were enrolled in our study met the clinical criteria for a hereditary syndrome, we believe that this aspect deserves particular attention.

Moreover, several cases of NETs have been described in patients with systemic lupus erythematosus (SLE) [28]. Indeed, a blockade of mTOR with N-acetylcysteine, Rapamycin and Sirolimus, another mTOR inhibitor, has shown promising results in both pre-clinical and clinical settings since the activity of mTOR has been found to be increased in lupus T cells [29,30,31,32]. Although none of the patients that were enrolled in our study fulfilled the criteria for a SLE diagnosis, we believe that, at least from a theoretical point of view, patients with NET and a simultaneous diagnosis of SLE could benefit from mTOR inhibitors. However, further in-depth studies are needed.

Our study has some limitations. First, the retrospective nature and low sample size could, at least in part, explain the imbalance of prognostic factors between the two groups and the differences between our and other series. Second, the heterogeneity of the series could mask biological differences, which could reflect into the variable expression of the mTOR pathway in NETs of different origin. Third, other components of mTOR pathway, such as Akt or 4E-BP1, deserve evaluation as potential predictive markers of Eve efficacy. Fourth, it would have been very interesting to repeat a biopsy after some weeks of treatment, in order to verify whether mTOR pathway expression can be considered to be a dynamic phenomenon and could change during treatment, thus predicting Eve efficacy. In this context, novel innovative approaches such as liquid biopsies would avoid invasive procedures and also deserve further investigations in NETs.

## 5. Conclusions

This study suggests that the activation of mTOR pathway can predict better efficacy of Eve in patients with NETs of various origin. However, these results warrant further prospective validation in larger and more homogeneous cohorts of patients.

## Figures and Tables

**Table 1:** Clinical-pathological characteristics.

	Group 1	Group 2		*p*-Value *
	**8 (33.3%)**	**16 (66.7%)**	**TOT (%)**	
**Gender**				
M	6 (75)	15 (93.75)	21 (87.5)	0.24
F	2 (25)	1 (6.25)	3 (12.5)	
**Age (years)**				
≤65	4 (50)	11 (68.75)	15 (62.5)	0.41
>65	4 (50)	5 (31.25)	9 (37.5)	
**Primary Site**				
Pancreas	0 (0)	14 (87.5)	14 (58.3)	<0.001
Ileum	5 (62.5)	1 (6.25)	6 (25)	
Others	3 (37.5)	1 (6.25)	4 (16.7)	
**Ki67 (%)**				
≤2	8 (100)	1 (6.25)	9 (37.5)	<0.001
>2	0 (0)	15 (93.75)	15 (62.5)	
**Grading**				
G1	8 (100)	1 (6.25)	9 (37.5)	<0.001
G2	0 (0)	14 (87.5)	14 (58.3)	
G3	0 (0)	1 (6.25)	1 (4.2)	
**Number ofmetastatic sites**				
0	0 (0)	2 (12.5)	2 (8.3)	
1	2 (25)	8 (50)	10 (41.7)	0.02
2	2 (25)	4 (25)	6 (25)	
3	3 (37.5)	1 (6.25)	4 (16.7)	
4	1 (12.5)	1 (6.25)	2 (8.3)	
**Secreting**				
Yes	4 (50)	2 (12.5)	6 (25)	0.3
No	4 (50)	14 (87.5)	18 (75)	
**Association with SSA**				
Yes	6 (75)	14 (87.5)	4 (16.7)	0.4
No	2 (25)	2(12.5)	20 (83.3)	
**Number of previous therapies**				
0	0 (0)	3 (18.7)	3 (12.5)	
1	0 (0)	4 (25)	4 (16.7)	
2	4 (50)	1 (6.25)	5 (20.8)	
3	3 (37.5)	5 (31.25)	8 (33.3)	0.11
4	0 (0)	2 (12.5)	2 (8.3)	
5	0 (0)	1 (6.25)	1 (4.2)	
6	0 (0)	0 (0)	0 (0)	
7	1 (12.5)	0 (0)	1 (4.2)	
Best objective response **				
Partial response	1 (16.7)	2 (12.5)	3 (13.6)	0.75
Stable disease	4 (66.7)	13 (81.25)	17 (77.3)	
Disease progression	1 (16.7)	1 (6.25)	2 (9.1)	
Number of subsequent therapies ***				
0	2 (28.6)	3 (18.75)	5 (21.7)	
1	5 (71.4)	3 (18.75)	8 (34.8)	
2	0 (0)	3 (18.75)	3 (13)	0.14
3	0 (0)	3 (18.75)	3 (13)	
4	0 (0)	3 (18.75)	3 (13)	
5	0 (0)	1 (6.25)	1 (4.4)	
**Primary removal**				
Yes	7 (87.5)	6 (37.5)	13 (54.2)	0.03
No	1 (12.5)	10 (62.5)	11 (45.8)	

(* Fisher’s exact test, statistical significance at a level of *p* < 0.05; ** evaluable in only 22 patients; *** evaluable only in 23 patients).

**Table 2:** Univariate Cox regression analysis.

		Univariate HR	CI 95%	*p*-value
	Age <65	1		
	Age ≥65	0.77	0.24–2.38	0.65
	pancreas	1		
	Ileum	0.61	0.12–2.95	0.54
**PFS**	Others	1.97	0.56–6.94	0.28
	G1	1		
	G2–G3	1.66	0.50–5.46	0.4
	p-mTOR/p-S6K negative	1		
	p-mTOR/p-S6K positive	0.74	0.22–2.43	0.62
	Age <65	1		
	Age ≥65	0.86	0.28–2.64	0.79
	pancreas	1		
	Ileum	0.64	0.13–3.13	0.59
**OS**	Others	3.71	0.97–14.12	0.05
	G1	1		
	G2-G3	1.64	0.50–5.35	0.4
	p-mTOR/p-S6K negative	1		
	p-mTOR/p-S6K positive	0.81	0.25–2.66	0.74
	Age <65	1		
	Age ≥65	0.99	0.29–3.32	0.98
	pancreas			
	Ileum	0.14	0.01–1.29	0.08
	Others	1.03	0.28–3.84	0.95
**OS from diagnosis of advanced diseases**	G1	1		
	G2–G3	5.38	1.12–25.78	0.03
	p-mTOR/p-S6K negative	1		
	p-mTOR/p-S6K positive	0.25	0.05–1,18	0.08

**Table 3:** Multivariate Cox regression analysis.

		Multivariate HR	CI 95%	*p*-value
**PFS**	p-mTOR/p-S6K negative	1		
	p-mTOR/p-S6K positive	0.37	0.06–2.29	0.28
**OS**	p-mTOR/p-S6K negative	1		
	p-mTOR/p-S6K positive	0.17	0.02–1.30	0.09
**OS from diagnosis of advanced disease**	p-mTOR/p-S6K negative	1		
	p-mTOR/p-S6K positive	0.19	0.02–1.50	0.11

## Abbreviations

Eve	Everolimus,
mTOR	mammalian target of Rapamicin,
NET	neuroendocrine tumor,
RR	response rate,
PFS	progression free survival,
OS	overall survival,
NENs	neuroendocrine neoplasms,
WHO	World Health Organization,
GEP	gastro-entero-pancreatic,
NECs	neuroendocrine carcinomas,
FKBP-12	FK506 binding protein-12,
mLST-8	mammalian lethal with SEC13 protein 8,
mTORC-1 and 2	mammalian target of Rapamicin Complex 1 and 2,
mSin-1	mammalian stress-activated protein kinase interacting protein 1
S6K	ribosomal protein S6 kinase,
4EBP1	eukaryotic initiation factor 4E-binding protein 1,
IGF-1	insulin growth factor 1,
IRS-1	insulin receptor substrate 1,
Myc	myelocytomatosis oncogene cellular homolog,
HIF-1	Hypoxia inducible factor 1,
pNETs	pancreatic neuroendocrine tumors,
DAB	3’-3-diaminobenzidine,
RECIST	response evaluation criteria in solid tumors,
SSA	somatostatin analog,
TACE	transarterial chemoembolization,
TSC-1 and 2	tuberous sclerosis complex 1 and 2,
PTEN	phosphatase and tensin homolog,
PI3K	Phosphatidylinositol 3-kinase
SLE	Systemic Lupus Erytematosus
